# Safety and efficacy of endovascular recanalization for symptomatic non-acute atherosclerotic intracranial large artery occlusion

**DOI:** 10.3389/fneur.2023.1144622

**Published:** 2023-04-27

**Authors:** Xi Chu, Yao Meng, Jun Zhang, Lili Sun, Hao Yin, Kaiyue Dong, Yingkun Chen, Yun Song, Meimei Zheng, Wei Wang, Wei Zhao, Ju Han

**Affiliations:** Department of Neurology, The First Affiliated Hospital of Shandong First Medical University, Shandong Provincial Qianfoshan Hospital, Jinan, China

**Keywords:** intracranial large artery occlusion, non-acute, interventional recanalization, complication, outcome

## Abstract

**Background and objective:**

The optimal treatment for patients with symptomatic non-acute atherosclerotic intracranial large artery occlusion (ILAO) despite medical treatment is not well established. We aimed to assess the safety, efficacy, and feasibility of angioplasty and stenting for these patients.

**Methods:**

A total of 251 consecutive patients with symptomatic non-acute atherosclerotic ILAO treated with interventional recanalization were retrospectively collected in our center from March 2015 to August 2021. The rate of successful recanalization, perioperative complications, and follow-up outcomes were evaluated.

**Results:**

Successful recanalization was achieved in 88.4% (222/251) of the patients. A total of 24 (24/251, 9.6%) symptomatic complications occurred among 251 procedures. In the 193 patients with clinical follow-up during 19.0 ± 14.7 months, 11 (11/193, 5.7%) patients developed ischemic stroke and four (4/193, 2.1%) patients developed transient ischemic attack (TIA). In the 106 patients with vascular imaging follow-up during 6.8 ± 6.6 months, seven (7/106, 6.6%) patients had restenosis and 10 (10/106, 9.4%) patients had reocclusion.

**Conclusion:**

This study suggests that interventional recanalization may be a feasible, basically safe, and an effective alternative in carefully selected patients with symptomatic non-acute atherosclerotic ILAO who have failed medical management.

## Introduction

Intracranial large artery occlusion (ILAO) attributed to advanced intracranial artery atherosclerosis is a very important cause of ischemic stroke, with a high rate of mortality, morbidity, and stroke recurrence. According to the Chinese Intracranial Atherosclerosis (CICAS) study, ~33.3% of ischemic stroke patients had intracranial large artery occlusion (ILAO), and recurrent stroke occurred in 7.27% of patients with total occlusion and in only 5.16% of patients with 70–99% stenosis during 1-year clinical follow-up (1).

A significant number of patients with acute ischemic stroke caused by ILAO do not receive thrombolytic therapy or mechanical thrombectomy in the acute phase for a variety of reasons and reached the non-acute phase. Patients with non-acute ILAO, especially those with hemodynamic compromise, have higher risks of recurrent stroke even with aggressive medical treatment. Moreover, long-term hypoperfusion may lead to cognitive impairment and poor quality of life (2). It remains unclear what is the optimal treatment for these patients.

Preliminary studies indicated that endovascular recanalization of non-acute ILAO may be an effective treatment approach for these patients (3–8). However, the clinical data are still lacking to assess the feasibility of this approach. In this study, we aimed to assess the safety and efficacy of endovascular recanalization for symptomatic atherosclerotic ILAO with a large amount of clinical data that were obtained from hundreds of endovascular recanalization for non-acute ILAO.

## Materials and methods

### Study population

This is a retrospective analysis of consecutive patients of non-acute atherosclerotic ILAO that underwent endovascular recanalization between March 2015 and August 2021 based on our prospective stroke database. Demographic, clinical, angiographic, and periprocedural data were collected. The protocol for this study was approved by our institutional ethics committee. Informed consent was obtained from all patients or their authorized surrogates.

The inclusion criteria were as follows: (1) patients aged between 18 and 80 years; (2) more than 24 h passed between the image-documented occlusion and intervention treatment; (3) ILAO diagnosed by magnetic resonance angiography (MRA) or computerized tomography angiography (CTA) and confirmed by digital subtraction angiography (DSA); (4) lesion locations included intracranial vertebral artery (ICVA), basilar artery (BA), intracranial internal carotid artery (ICICA), and middle cerebral artery (MCA); (5) recurrent ischemic stroke or transient ischemic attack (TIA) related to the occlusive arteries despite aggressive medical treatment and preoperative arterial spin labeling (ASL) revealing hypoperfusion in the target artery territory; and (6) the American Society of Interventional and Therapeutic Neuroradiology/Society of Interventional Radiology Collateral Flow Grading System score being <3 on DSA.

The exclusion criteria were as follows: (1) non-atherosclerotic occlusions, such as arterial dissection, moyamoya disease, and vasculitis disease; (2) clinical symptoms were stable with aggressive medical treatment; (3) patients suffering from other severe diseases and life expectancy <1 year; and (4) contraindications to operation, such as known allergy or contraindication to aspirin, clopidogrel, heparin, or anesthesia.

### Procedure

All operations were performed via the percutaneous transfemoral route under general anesthesia by a highly experienced neuroradiologist. The details of the intervention procedure were described previously (9–11). Conventional balloons were applied for balloon angioplasty, and drug-coated balloons (DCB) (SeQuent Please, B. Braun, Germany) were used in subsequent studies after conventional balloon angioplasty in an attempt to reduce intimal hyperplasia (12, 13) and the risk of restenosis or reocclusion. Remedial stenting was performed when residual stenosis exceeding 50% or vessel dissection occurred after balloon angioplasty. Patients with heavy thrombus loads were treated with catheter aspiration or stent retrieval device, or with low-dose tirofiban or urokinase through the catheter.

Angiography was applied to evaluate distal blood perfusion of recanalized vessels according to the Thrombolysis in Cerebral Infarction (TICI) grading system. TICI ≥ 2b at the end of the procedure was considered to be technical success. An immediate CT scan of the brain was conducted following the procedure to exclude cerebral hemorrhage.

### Perioperative management and follow-up

Dual antiplatelet therapy (DAPT) with aspirin (100 mg) and clopidogrel (75 mg) was initiated at least 5 days before intervention. Thromboelastography was performed voluntarily to evaluate the platelet reactivity. For patients at high risk of thrombosis, clopidogrel was increased to 100 mg daily or using ticagrelor 90 mg twice a day instead. In patients at high risk of bleeding, aspirin or clopidogrel dosage was reduced to 50 mg, or indocybufen 100 mg twice a day was substituted for aspirin. After intervention recanalization, blood pressure was strictly monitored and controlled to prevent hyperperfusion syndrome. DAPT was maintained for 6 months for patients with stenting and 3 months for patients with angioplasty and then followed by lifelong aspirin or clopidogrel monotherapy. The patients were scheduled to perform DSA at 3–6 months voluntarily. The main outcomes were ischemic stroke, TIA, rate of restenosis/occlusion, and death attributed to stroke in subsequent follow-up. Restenosis and reocclusion were defined as > 50% stenosis within or immediately adjacent (within 5 mm) of the treated segment and >20% absolute luminal loss (14) or total occlusion of the target artery segment with imaging follow-up, respectively. Symptomatic restenosis and reocclusion were defined as restenosis or reocclusion accompanied by ischemic symptoms in the offense vessel territory. Clinical and imaging outcomes were reviewed by two investigators. Disagreements were settled by consensus.

### Statistical analysis

Continuous data were expressed as mean ± SD or as the median with interquartile range (IQR), whereas categorical data were presented as numbers and percentages. The Wilcoxon rank sum test was used to compare the mRS scores at discharge and 6 months with that before treatment. A *p*-value of <0.05 was considered to be statistically significant. Statistical analysis was performed using SPSS version 26.0 for Windows (SPSS Inc., Chicago, USA).

## Results

**Figure 1:**
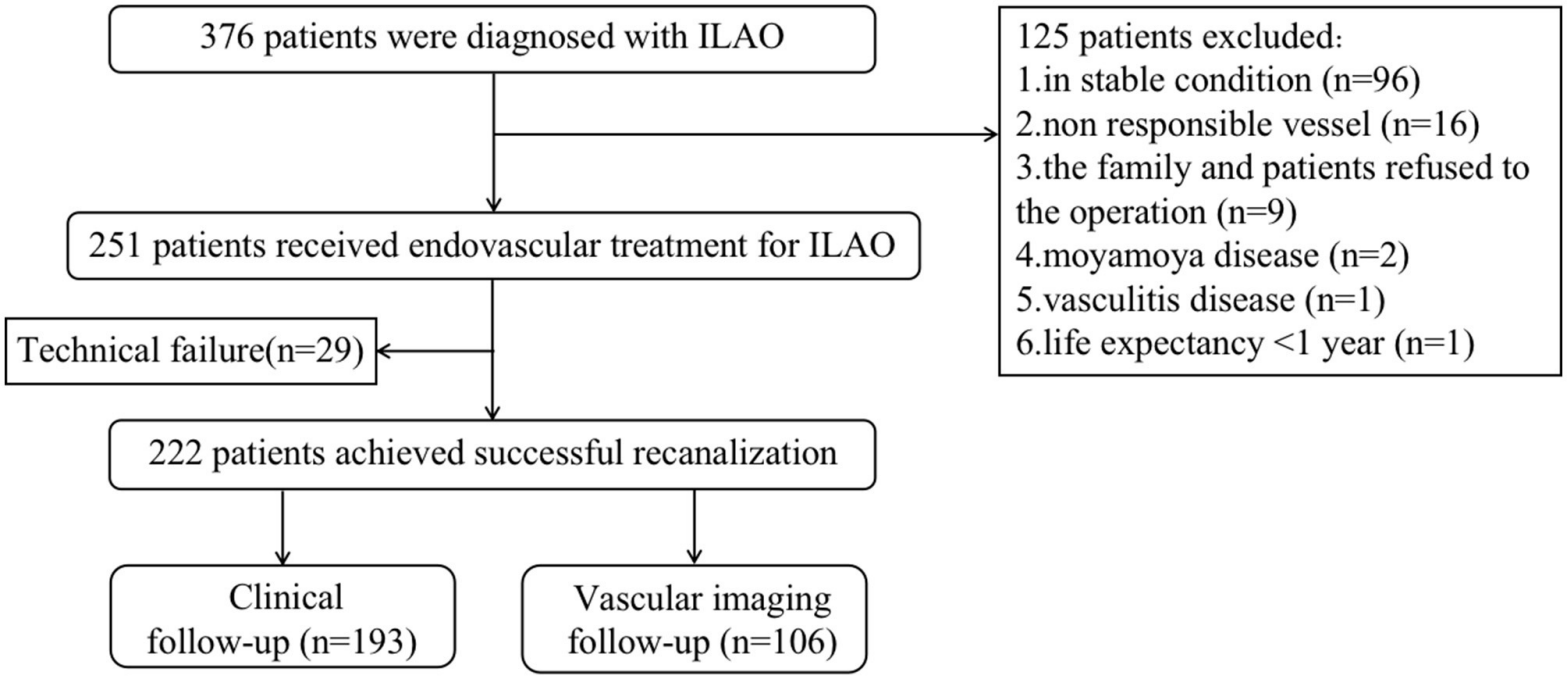
Flowchart of the study. ILAO, intracranial large artery occlusion.

A total of 381 patients diagnosed with ILAO were enrolled between March 2015 and August 2021. A total of 130 patients were excluded. At last, 251 patients were finally enrolled. The flowchart of the study is illustrated in Figure 1. There were 47 lesions in ICICA, 122 lesions in MCA, 40 lesions in ICVA, and 42 lesions in BA (Table 1). Baseline demographic and clinical variables are shown in Table 1. Hypertension (*N* = 197, 78.5%), diabetes mellitus (*N* = 87, 34.7%), smoking (*N* = 109, 43.4%), and drinking (*N* = 90, 35.9%) were the most common risk factors. The median pre-treatment NIHSS score was 3.0 (IQR, 1.0–7.0), and the median pre-treatment mRS score was 2.0 (IQR, 1.0–3.0). The median time from symptom onset to treatment was 27.0 days (IQR, 16.0–53.0 days), and the median time from occlusion confirmed to treatment was 12.0 days (IQR, 6.0–24.0 days) in all patients.

**Table 1:** Patients' baseline demographic and clinical variables.

**Variables**	**Overall (*n* = 251)**	**ICICA** **(*n* = 47)**	**MCA (*n* = 122)**	**ICVA** **(*n* = 40)**	**BA (*n* = 42)**
Sex, male	167 (66.5%)	26 (55.3%)	73 (59.8%)	35 (87.5%)	33 (78.6%)
Age (years), mean ± SD	58.7 ± 9.4	58.0 ± 9.2	58.8 ± 9.7	60.7 ± 7.8	57.3 ± 9.9
Hypertension	197 (78.5%)	33 (70.2%)	96 (78.7%)	33 (82.5%)	35 (83.3%)
Diabetes mellitus	87 (34.7%)	19 (40.4%)	40 (32.8%)	19 (47.5%)	9 (21.4%)
Coronary artery disease	45 (17.9%)	8 (17.0%)	24 (19.7%)	7 (17.5%)	6 (14.3%)
Hyperlipidemia	45 (17.9%)	8 (17.0%)	27 (22.1%)	6 (15.0%)	4 (9.5%)
Atrial fibrillation	6 (2.4%)	2 (4.3%)	2 (1.6%)	1 (2.5%)	1 (2.4%)
Smoking	109 (43.4%)	17 (36.2%)	45 (36.9%)	23 (57.5%)	24 (57.1%)
Drinking	90 (35.9%)	14 (29.8%)	33 (27.0%)	20 (50.0%)	23 (54.8%)
Pre-treatment NIHSS score, median (IQR)	3.0 (1.0–7.0)	2.0 (1.0–6.0)	3.0 (1.0–6.0)	4.0 (0.0–6.8)	4.0 (1.0–7.0)
Pre-treatment mRS score, median (IQR)	2.0 (1.0–3.0)	2.0 (1.0–4.0)	2.0 (1.0–3.0)	2.0 (1.0–3.0)	2.5 (1.0–4.0)
Symptom onset to treatment (days), median (IQR)	27.0 (16.0–53.0)	27.0 (19.0–70.0)	28.0 (17.0–56.3)	23.5 (16.0–46.8)	25.5 (16.0–56.0)
Occlusion confirmed to treatment (days), median (IQR)	12.0 (6.0–24.0)	18.0 (6.0–36.0)	14.0 (7.0–28.0)	12.5 (6.0–19.0)	7.0 (2.8–12.0)

ICICA, intracranial internal carotid artery; MCA, middle cerebral artery; ICVA, intracranial vertebral artery; BA, basilar artery; NIHSS, National Institutes of Health Stroke Scale; mRS, modified Rankin Scale; SD, standard deviation; IQR, interquartile range.

**Figure 2:**
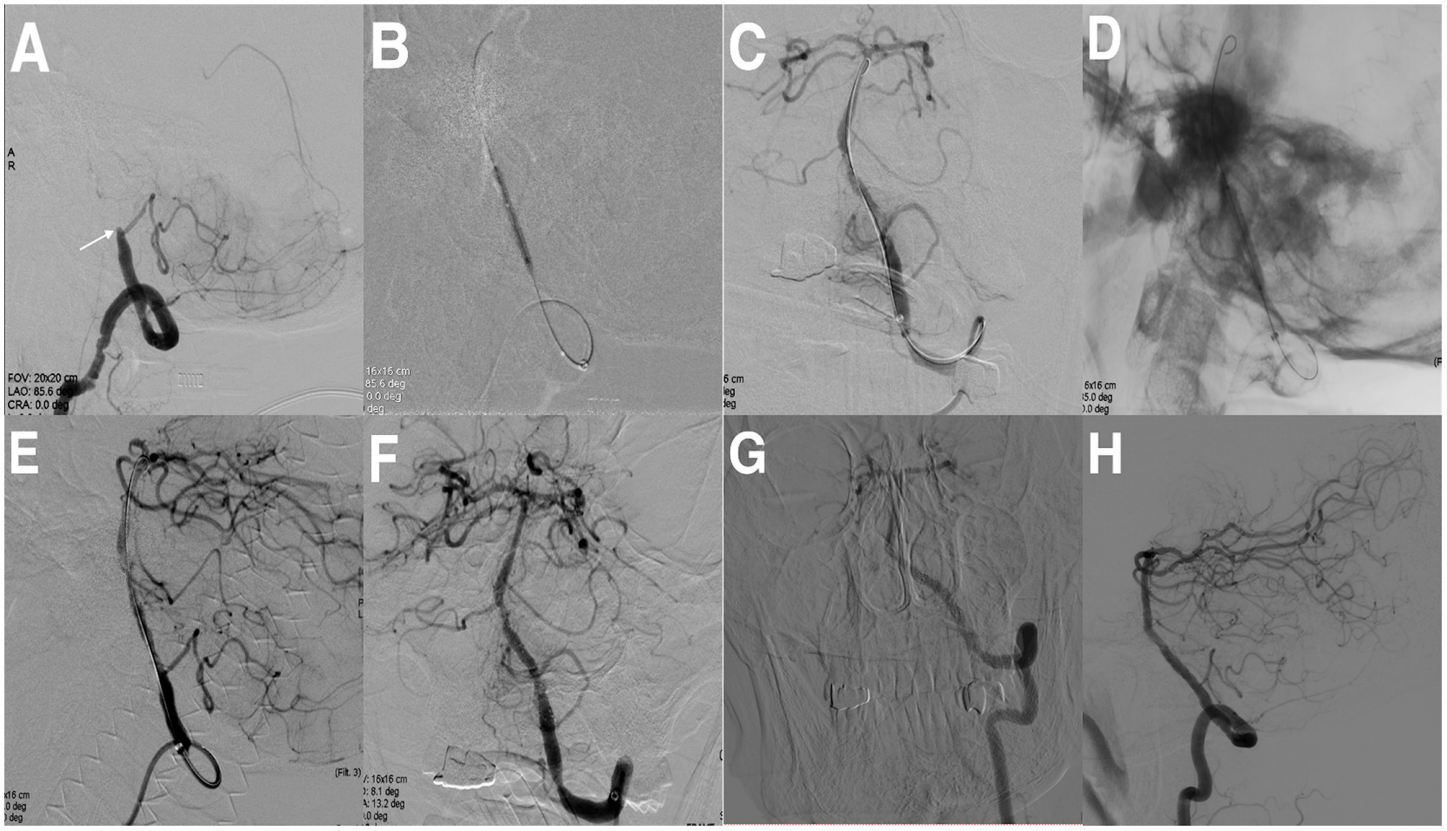
Cerebral angiographic results of drug-coated balloon (DCB) dilatation and stenting implantation for intracranial vertebral artery (ICVO) during the procedure and follow-up. **(A)** ICVO (the arrow indicates the occlusion site). **(B)** Predilation with a conventional balloon. **(C)** Angiographic result after predilatation with a conventional balloon. **(D)** Predilatation with DCB. **(E)** Angiographic result after predilatation with DCB. **(F)** Angiographic result after stenting implantation. **(G, H)** Angiographic result at the 8-month follow-up with a patent vertebral artery.

As shown in Table 2, 88.4% (222/251) of the patients achieved successful recanalization, of which there were 42 patients (89.4%) in the ICICA, 112 patients (91.8%) in the MCA, 34 patients (85.0%) in the ICVA, and 34 patients (81.0%) in the BA. An overview of 222 patients' treatment modalities is presented in Table 2. In total, 21 patients (9.5%) received conventional balloon angioplasty (CBA), 46 patients (20.7%) received CBA and stent implantation, 106 patients (47.7%) received CBA and drug-coated balloon angioplasty (DCBA), and 49 patients (22.1%) received CBA, DCBA, and stent implantation. An illustrative case is shown in Figure 2. The median residual stenosis was 0.0% (IQR, 0.0–20.0%) in patients who were successfully recanalized. In all patients, postrecanalization angiography demonstrated TICI grade 3 in 187 patients (74.5%) patients, TICI grade 2b in 35 patients (13.9%), TICI grade 2a in five patients (2.0%), and TICI grade 1 in four patients (1.6%).

**Table 2:** Treatment modalities and pre-discharge outcomes of the patients.

**Variables**	**Overall *N* = 251**	**ICICA** ***N* = 47**	**MCA *N* = 122**	**ICVA** ***N* = 40**	**BA *N* = 42**
**Technical success**	222 (88.4%)	42 (89.4%)	112 (91.8%)	34 (85.0%)	34 (81.0%)
CBA	21 (9.5%)	4 (9.5%)	10 (8.9%)	3 (8.8%)	4 (11.8%)
CBA + stenting	46 (20.7%)	7 (16.7%)	20 (17.9%)	9 (26.5%)	10 (29.4%)
CBA + DCBA	106 (47.7%)	24 (57.1%)	62 (55.4%)	8 (23.5%)	12 (35.3%)
CBA + DCBA + stenting	49 (22.1%)	7 (16.7%)	20 (17.9%)	14 (41.2%)	8 (23.5%)
**Postprocedural perfusion**					
TICI = 3	187 (74.5%)	26 (55.3%)	96 (78.7%)	31 (77.5%)	34 (81.0%)
TICI = 2b	35 (13.9%)	16 (34.0%)	16 (13.1%)	3 (7.5%)	0 (0.0%)
TICI = 2a	5 (2.0%)	1 (2.1%)	2 (1.6%)	1 (2.5%)	1 (2.4%)
TICI = 1	4 (1.6%)	0 (0.0%)	1(0.8%)	0 (0.0%)	3 (7.1%)
Residual stenosis (%), median (IQR)	0.0 (0.0–20.0)	0.0 (0.0–40.0)	0.0 (0.0–0.0)	0.0 (0.0–21.3)	0.0 (0.0–20.0)
**Periprocedural complications**	34 (13.5%)	6 (12.8%)	18 (14.8%)	4 (10.0%)	6 (14.3%)
Symptomatic complications	24 (9.6%)	4 (8.5%)	12 (9.8%)	3 (7.5%)	5 (11.9%)
Vascular perforation	4 (1.6%)	1 (2.1%)	3 (2.5%)	0 (0.0%)	0 (0.0%)
Acute thrombosis	4 (1.6%)	1 (2.1%)	2 (1.6%)	0 (0.0%)	1 (2.4%)
Symptomatic ICH	15 (6.0%)	1 (2.1%)	12 (9.8%)	1 (2.5%)	1 (2.4%)
Asymptomatic ICH	1 (0.4%)	0 (0.0%)	1 (0.8%)	0 (0.0%)	0 (0.0%)
Distal embolization	4 (1.6%)	2 (4.3%)	0 (0.0%)	1 (2.5%)	1 (2.4%)
Perforator stroke	5 (2.0%)	0 (0.0%)	0 (0.0%)	2 (5.0%)	3 (7.1%)
Stroke^*^	1 (0.4%)	1 (2.1%)	0 (0.0%)	0 (0.0%)	0 (0.0%)
Death	4 (1.6%)	0 (0.0%)	2 (1.6%)	0 (0.0%)	2 (4.8%)

ICICA, intracranial internal carotid artery; MCA, middle cerebral artery; ICVA, intracranial vertebral artery; BA, basilar artery; CBA, Conventional Balloon Angioplasty; DCBA, Drug-Coated Balloon Angioplasty; TICI, Thrombolysis in Cerebral Infarction; IQR, interquartile range; ICH, intracranial hemorrhage; Stroke^*^, defined as ischemic stroke of unknown cause; Symptomatic complications, defined as perioperative complications with clinical symptoms.

In our study, 29 patients experienced technical failure: 20 cases failed because of the micro-guidewire failing to pass through the occlusion segment; two cases failed because of vascular perforation, and the operation was terminated for safety reasons; one patient failed because of reocclusion after balloon dilatation and stent implantation. The remaining six patients were not classified as technical success because of TICI <2b despite partial recanalization being achieved.

**Figure 3:**
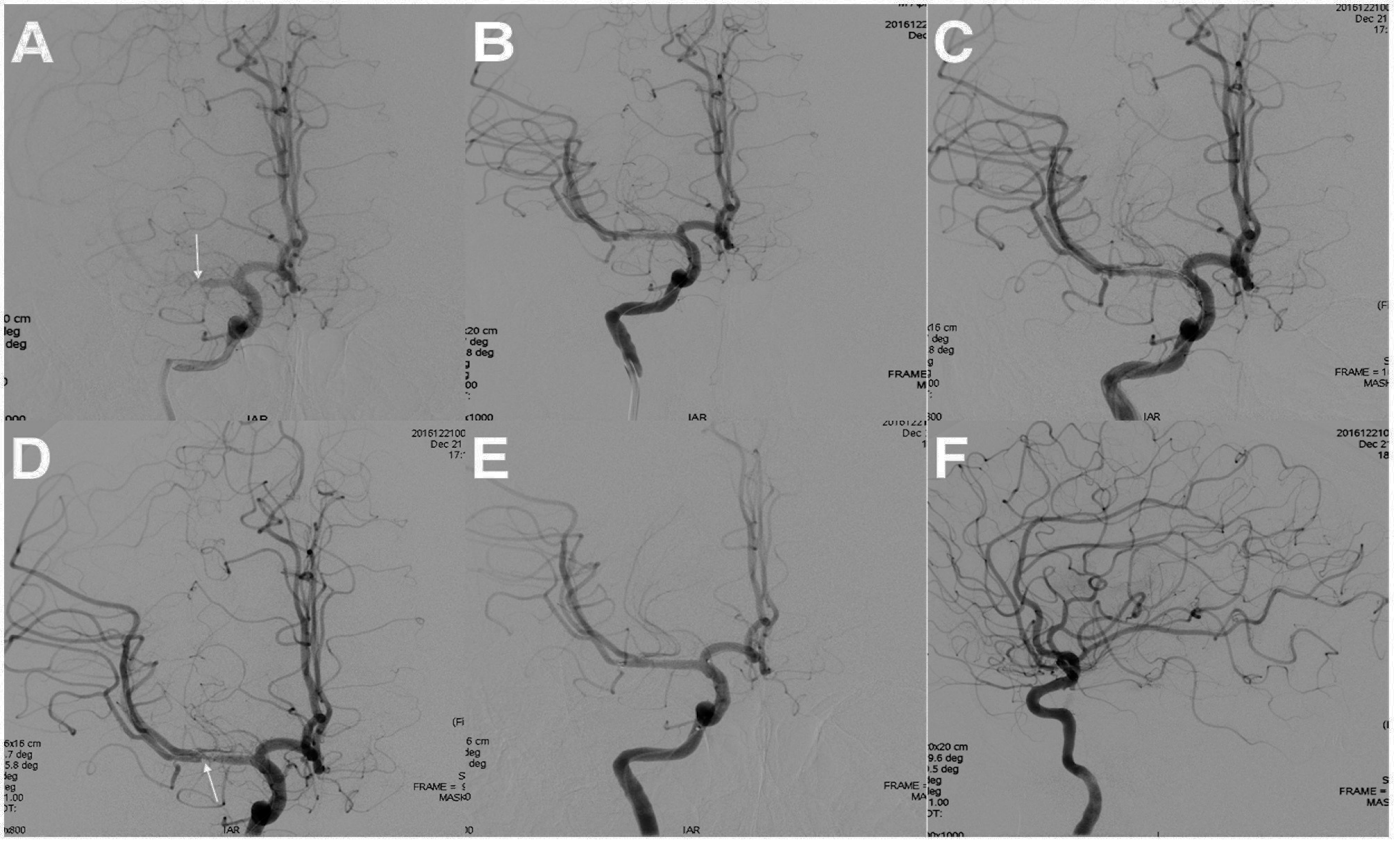
Intraoperative angiographic results in a patient with acute thrombosis. **(A)** Middle cerebral artery occlusion (the arrow indicates the occlusion site). **(B)** Angiographic result after predilation with a conventional balloon. **(C)** Angiographic result after stenting implantation. **(D)** Angiographic result after 10 min of stent implantation (the arrow indicates acute thrombosis). **(E, F)** Angiographic result after combined application of tirofiban *via* artery and vein.

Periprocedural complications occurred in 34 patients (13.5%) including vascular perforation, acute thrombosis, hemorrhagic transformation, distal embolization, and perforator stroke (Table 2). Vascular perforation occurred in four patients (1.6%) (three in the MCA and one in the ICICA), without symptomatic intracranial hemorrhage after coil packing. Acute thrombosis occurred in four patients (1.6%) (two in the MCA, one in the ICICA, and one in the BA), and the acute thrombosis disappeared after the application of tirofiban via artery and vein. Figure 3 is a representative case. Intracranial hemorrhage occurred in 16 (6.4%) patients after recanalization of the target lesion (13 in the MCA, one in the ICICA, one in the ICVA, and one in the BA): one patient experienced asymptomatic intracranial hemorrhage; eight patients experienced headache or dizziness, and the symptoms disappeared after medication or trepanation and drainage; four patients experienced limb weakness and/or slurred speech, and neurological deficits were left at discharge after drug treatment and/or decompressive craniectomy; and three patients died. Distal embolization occurred in four patients (1.6%) (two in the ICICA, one in the BA, and one in the ICVA): one case was without adverse consequences through successful aspiration; one case had numbness of fingers and soles; and two cases had blurred vision on the ipsilateral to the recanalized vascular. Perforator strokes were observed in five patients (2.0%) (three in the BA and two in the ICVA). One died, and the rest were discharged with neurological deficits. In addition, there was a patient with ischemic stroke of unknown cause, which may be associated with severe residual stenosis. To sum up, 24 (9.6%) patients had symptomatic periprocedural complications, of which 12 patients' clinical symptoms almost disappeared at discharge.

Follow-up clinical and angiographic outcomes of successfully treated patients are shown in Table 3. A total of 193 cases of successful recanalization were followed up clinically. During the clinical follow-up period of 19.0 ± 14.7 months, the median mRS score was 1.0 (IQR, 0.0–3.0). In total, 11 (11/193, 5.7%) patients experienced ischemic stroke and four patients (4/193, 2.1%) experienced TIA. During the clinical follow-up period, three deaths (3/193, 1.6%) were attributed to stroke, but two patients did not have follow-up imaging. During the vessel imaging follow-up period of 6.8 ± 6.6 months, restenosis occurred in seven patients (7/106, 6.6%) and two patients (2/106, 1.9%) presented with symptomatic restenosis. Reocclusion occurred in 10 patients (10/106, 9.4%) and 4 patients (4/106, 3.8%) presented with symptomatic reocclusion.

**Table 3:** Follow-up clinical and angiographic outcomes of successfully treated patients.

**Variables**	**Overall**	**ICICA**	**MCA**	**ICVA**	**BA**
**Clinical outcomes**					
Number of clinical follow-up	193	40	99	25	29
Follow-up time (months), mean (SD)	19.0 ± 14.7	20.5 ± 14.5	17.7 ± 14.4	20.6 ± 14.7	20.2 ± 16.5
mRS score at last follow-up, median (IQR)	1.0 (0.0–3.0)	1.0 (0.0–2.8)	1.0 (0.0–3.0)	1.0 (0.0–2.0)	2.0 (0.5–3.0)
Ischemic stroke	11 (5.7%)	1 (2.5%)	7 (7.1%)	0 (0.0%)	3 (10.3%)
Hemorrhagic stroke	1 (0.5%)	0 (0.0%)	1 (1.0%)	0 (0.0%)	0 (0.0%)
TIA	4 (2.1%)	1 (2.5%)	1 (1.0%)	1 (4%)	1 (3.4%)
Stroke-related death	3 (1.6%)	0 (0.0%)	2 (2.0%)	0 (0.0%)	1 (3.4%)
**Angiographic outcomes**					
Number of imaging follow-up	106	22	55	14	15
Imaging follow-up time (months), mean (SD)	6.8 ± 6.6	8.0 ± 8.1	5.9 ± 6.2	7.6 ± 5.4	7.7 ± 6.5
Restenosis on follow-up image	7 (6.6%)	1 (4.5%)	5 (9.1%)	0 (0.0%)	1 (6.7%)
Symptomatic restenosis	2 (1.9%)	0 (0.0%)	1 (1.8%)	0 (0.0%)	1 (6.7%)
Reocclusion on follow-up image	10 (9.4%)	1 (4.5%)	7 (12.7%)	1 (7.1%)	1 (6.7%)
Symptomatic reocclusion	4 (3.8%)	1(4.5%)	2 (3.6%)	0 (0.0%)	1 (6.7%)

ICICA, intracranial internal carotid artery; MCA, middle cerebral artery; ICVA, intracranial vertebral artery; BA, basilar artery; mRS, modified Rankin Scale; TIA, transient ischemic attack; IQR, interquartile range; SD, standard deviation.

A total of 180 patients were followed up at 6 months. mRS scores of all patients and subgroups of ICICA, MCA, ICVA, and BA at discharge and 6 months after the operation were significantly lower than before the operation, as shown in Table 4.

**Table 4:** Comparative analysis of mRS scores at different stages of successfully treated patients.

		**Pre-treatment**	**At discharge**	**6-month follow-up**
Overall *N* = 180	mRS score, median (IQR)	2.0 (1.0–4.0)	2.0 (1.0–3.0)	1.0 (0.0–3.0)
	Z-statistic		−6.197	−7.867
	*P*-value		<0.001	<0.001
ICICA *N* = 36	mRS score, median (IQR)	1.5 (1.0–3.8)	1.0 (1.0–2.8)	1.0 (0.0–2.0)
	Z-statistic		−3.217	−3.981
	*P*-value		0.001	<0.001
MCA *N* = 93	mRS score, median (IQR)	2.0 (1.0–4.0)	2.0 (1.0–3.0)	1.0 (0.0–3.0)
	Z-statistic		−3.890	−4.878
	*P*-value		<0.001	<0.001
ICVA *N* = 25	mRS score, median (IQR)	3.0 (1.0–3.0)	2.0 (1.0–3.0)	1.0 (0.0–2.0)
	Z-statistic		−2.950	−3.501
	*P*-value		0.003	<0.001
BA *N* = 26	mRS score, median (IQR)	3.0 (1.0–4.0)	2.0 (1.0–4.0)	2.0 (1.0–3.0)
	Z-statistic		−2.121	−3.402
	*P*-value			0.034	0.001

Z-statistic shows comparison to pre-treatment mRS score; ICICA, intracranial internal carotid artery; MCA, middle cerebral artery; ICVA, intracranial vertebral artery; BA, basilar artery; mRS, modified Rankin Scale; IQR, interquartile range.

## Discussion

A significant number of patients with symptomatic atherosclerotic non-acute ILAO treated with aggressive medical therapy were still hemodynamically unstable and experienced recurrent and progressive ischemic events. This study provides a summary of our center's clinical experience in intervention recanalization for this disease. To the best of our knowledge, this is the largest series of cases ever reported.

The success rate of interventional recanalization varies significantly among centers (3–8), which may be related to patient selection, the ability of the operator, and the different definitions of technical success. The technical difficulty in recanalization of ILAO is the micro-guidewire passing through the occluded segment, which is related to the nature, length, and shape of the occluded segment's vessels. First, the intracranial vessels are thinner and more fragile than the large cervical vessels. Second, different from stenotic lesions, distal blood vessels of the occluded artery are invisible, and the length of the occluded segment and anatomical outline of the blood vessels are difficult to determine. Third, its main component is fibrous calcified plaque, which is harder than acute thrombus (5). These increase the difficulty of the operation. In recent years, high-resolution black-blood MRI has been used to study the ILAO (15) and could direct the endovascular recanalization of this disease. It could help to evaluate whether the occlusion is acute or subacute thrombosis or chronic plaque formation and evaluate the length and contortion of vessels. In addition, when the DSA shows a tapered end, the micro-guidewire tends to pass through the occlusion more easily (7).

Perioperative complications in this study included vascular perforation, acute thrombosis, hemorrhagic transformation, distal embolization, and perforator stroke. In this study, patients with perforation and acute thrombosis generally did not suffer from neurological deficits after appropriate treatments. Based on the findings of our study, perforator strokes could result in serious adverse clinical outcomes, but the incidence is relatively low. All perforator strokes occurred in the posterior circulation, which is consistent with the previous study of Gao et al. (16) on the recanalization of basilar artery occlusion. It is worth mentioning that intracranial hemorrhage was associated with significant disability and mortality. The hemorrhagic transformation risk was higher for MCA occlusion recanalization in this study. Further research is necessary to improve the safety of this technology in the prevention and management of perforator strokes, distal embolization, and hemorrhagic transformation.

The natural course of symptomatic atherosclerotic ILAO with hemodynamic impairment is poor (the annual incidence of ipsilateral stroke is 23.7%) (17). The event rate (5.7% of patients had ischemic stroke and 2.1% had TIA) remained relatively low over the average 19.0-month clinical follow-up period in this study. These patients at high risk of stroke recurrence may benefit from endovascular recanalization, as found in this and previous studies (3, 4, 18). In addition, the mRS scores of the 180 patients followed up 6 months after the operation were significantly lower than that before the operation, which was statistically significant (*P* < 0.05). Perfusion was restored after endovascular recanalization for these patients, and the clinical outcome was improved. Patients with clinical symptoms due to insufficient perfusion may benefit from endovascular recanalization. Further research is needed to compare this treatment with drug treatment alone and determine which patients are the best candidates for this treatment.

Over the average 6.8-month vessel imaging follow-up period, seven patients (6.6%) had restenosis and 10 patients (9.4%) had reocclusion among the 106 patients. Patients with successful recanalization were required to undergo DSA 3–6 months after the procedure, but many of them were unwilling to undergo DSA due to stable symptoms, and there may be patient selection bias. Thus, the real rate of restenosis and reocclusion may be lower than the current rate. The incidence of restenosis or reocclusion is a major factor affecting the long-term curative effect of the procedure. Our center's research initially showed that DCB dilation can effectively lower restenosis degree and total restenosis risk compared with conventionally only stenting angioplasty (19). Moreover, DCB dilation in the intracranial artery to treat in-stent restenosis is feasible and safe (20). A large-scale clinical trial is warranted to further evaluate the safety and effectiveness of this treatment.

However, there are some limitations to our study. First, this is a retrospective study, and we did not compare this cohort with non-acute atherosclerotic ILAO patients who did not undergo the interventional recanalization, so further large-scale randomized controlled trials are needed to confirm the safety and effectiveness of interventional recanalization for the treatment of non-acute symptomatic atherosclerotic ILAO. Second, the small sample size of follow-up imaging data in patients may limit the evaluation of the overall restenosis and reocclusion rate.

## Conclusion

In conclusion, patients with symptomatic non-acute atherosclerotic ILAO presented with recurrent and progressive ischemic events despite aggressive medical therapy. Interventional recanalization could offer an alternative option for this type of disease with basic safety and clinical efficacy. However, it should be warned that perforator stroke and reperfusion hemorrhage can result in severe disability and even death. Therefore, more rigorous patient selection, better prevention and management of perioperative complications, and more advanced technical materials and strategies are needed to improve the safety and efficacy of the technique, which can benefit patients even more.

## Data availability statement

The original contributions presented in the study are included in the article/supplementary material, further inquiries can be directed to the corresponding authors.

## Ethics statement

The studies involving human participants were reviewed and approved by the Medical Ethics Committee of Shandong Provincial Qianfoshan Hospital. The patients/participants provided their written informed consent to participate in this study.

## Author contributions

XC contributed to the acquisition, analysis, and interpretation of the data, drafting the article, and revising the content. YM, JZ, and LS contributed to the analysis of the data and the revision of content. HY, KD, and YC contributed to the analysis of the data. YS, MZ, and WW contributed to the statistical analysis. WZ and JH contributed to the conception and design of the study, acquisition and analysis of the data, drafting of the article, and revision of the content. All authors approved the contents of the article and also agree to be accountable for all aspects of the study submitted for publication.
